# An Atypical Presentation of Tuberculosis With the Involvement of the Thoracic Aorta

**DOI:** 10.7759/cureus.106797

**Published:** 2026-04-10

**Authors:** Sean Namazi, Cindy Hou

**Affiliations:** 1 Medical Education, Rowan-Virtua School of Osteopathic Medicine, Stratford, USA; 2 Infectious Disease, Jefferson Health, Cherry Hill, USA

**Keywords:** recurrent hemoptysis, thoracic aorta, thoracic endovascular aortic repair, tuberculosis, tuberculous aortitis

## Abstract

Tuberculous aortitis (TA) is a rare complication of *Mycobacterium tuberculosis* (TB) characterized by persistent inflammatory infiltration and weakening of the aortic wall. The disease most commonly results from contiguous spread from adjacent infected tissue and can lead to life-threatening complications, notably pseudoaneurysm formation and aortic rupture. The present case describes a 66-year-old man with sudden-onset back pain and hemoptysis. He was initially found to have a penetrating aortic ulcer (PAU), but subsequent radiographic imaging and recurrent hospitalizations later revealed aortic findings more consistent with TA. This report highlights the rarity of TA co-occurring with a PAU and emphasizes existing treatment plans for this clinical scenario.

## Introduction

Tuberculosis (TB) is a widely known infectious disease with a broad spectrum of fatal complications. Before the COVID-19 pandemic, it was recognized as one of the most lethal diseases worldwide, contributing substantially to widespread morbidity and mortality among marginalized sections of the population [1].

In 2023, the United States reported 9,615 TB cases, a 16% increase from 2022. The incidence rose from 2.5 to 2.9 per 100,000 people [2]. According to the World Health Organization, tuberculosis is viewed as a global public health emergency and the leading cause of death in people with associated immunodeficiency, particularly HIV/AIDS [1,3].

The pathophysiology of TB is characterized by granulomatous inflammation with caseous necrosis and can often result in systemic signs, including fever, night sweats, and weight loss, with blood-stained chronic cough as a typical late manifestation of the disease [4]. In rare cases, this inflammatory process can result in weakening of the aortic vessel wall through tissue necrosis and degradation of structural proteins, leading to the development of tuberculous aortitis (TA) [5].

To our knowledge, fewer than 100 cases of TA have been reported in the medical literature. The disease onset is often insidious, presenting as a delay between symptom onset and diagnosis, with vascular manifestations unlike typical TB, including asymmetric blood pressure, claudication, and diminished pulses [4].

One of the feared complications of TA is pseudoaneurysm formation, which carries a high risk of spontaneous rupture within three months. Rupture is associated with markedly worse outcomes, with mortality approaching 35%-40% overall and 25%-30% aneurysm-related mortality within two years compared with substantially lower mortality among non-ruptured aneurysms [4].

The present case describes TB complicated by TA with a mycotic aneurysm that presented initially as a penetrating atherosclerotic ulcer (PAU). A PAU is an ulceration of underlying atherosclerotic plaque with focal disruption of the aortic intima [6]. This case was unique in that radiographic imaging showed minimal atherosclerotic plaque, which is atypical for a PAU, and his subsequent clinical progression soon provided evidence of TA following the initial diagnosis. To date, no cases of PAU co-occurring with TA have been reported.

## Case presentation

A 66-year-old Filipino man presented with sudden-onset interscapular pain for several days, followed by episodes of hemoptysis. His review of systems was negative for any fever, chills, night sweats, weight loss, or shortness of breath.

His past medical history includes latent TB treatment in 2006 and a motor vehicle accident (MVA) in 2016. He had no past surgical history. Travel history was notable for a return from the Philippines three weeks before his presentation. He denied tobacco, alcohol, or illicit drug use. 

The patient was afebrile with a heart rate of 120 beats per minute, blood pressure of 123/80 mmHg, respiratory rate of 18 breaths per minute, and an oxygen saturation of 95% on room air. His height was 5’6’ and weight 128 pounds, with a BMI of 20.7. On physical examination, the patient appeared thin and in no acute distress. Pulmonary effort and breath sounds were unremarkable.

Upon laboratory investigation, the patient’s relative monocyte count, bilirubin (direct and indirect), alkaline phosphatase (ALP), aspartate aminotransferase (AST), and alanine aminotransferase (ALT) were mildly elevated; otherwise, all other laboratory findings were unremarkable (Table 1).

**Table 1:** Summary of the laboratory findings and reference values at admission. Notable findings include relative monocyte count, bilirubin (direct and indirect), ALP, AST, and ALT Abbreviations: ALP, alkaline phosphatase; WBC, white blood cell count; RBC, red blood cell count; Hgb, hemoglobin; Hct, hematocrit; MCV, mean corpuscular volume; MCH, mean corpuscular hemoglobin; MCHC, mean corpuscular hemoglobin concentration; RDW, red cell distribution width; MPV, mean platelet volume; CO₂, serum bicarbonate; eGFR, estimated glomerular filtration rate; AST, aspartate aminotransferase; ALT, alanine aminotransferase; INR, international normalized ratio; PT, prothrombin time; BNP, B-type natriuretic peptide. Arrows indicate values above (↑) or below (↓) the reference range.

Laboratory Marker	Reference Range	Patient's Values
White Blood Cell Count	3.7-10.5x10^9^/L	6.3x10^9^/L
Red Blood Cell Count	4.10-5.45x10^12^/L	5.71x10^12^/L ↑
Hemoglobin	12.8-16.3 g/dL	17.3 g/dL↑
Hct	37.1-48.5%	51.5% ↑
MCV	80-96.5 fL	90.2 fL
MCH	27.0-33.1 pg	30.3 pg
MCHC	32.5-35.5 g/dL	33.6 g/dL
RDW	12.5-16.0%	13.5%
Platelet	150-400x10^9^/L	309x10^9^/L
MPV	7.2-10.6 fL	6.1 fL ↓
Neutrophils %	40.0-73.0%	66.9%
Lymphocytes %	20.0-44.0%	17.1% ↓
Monocytes %	3.0-13.0%	14.9% ↑
Eosinophils %	0.0-6.0%	0.4%
Basophils %	0.0-3.0 %	0.7%
Neutrophils Absolute	1.60-8.00x10^9^/L	4.2x10^9^/L
Lymphocytes Absolute	0.80-4.80 B/L	1.1x10^9^/L
Monocytes Absolute	0.10-1.40x10^9^/L	0.9x10^9^/L
Eosinophils Absolute	0.00-0.70x10^9^/L	0x10^9^/L
Basophils Absolute	0.00-0.30x10^9^/L	0x10^9^/L
Nucleated RBC	0.0-0.5%	0%
Sodium	133-145 mmol/L	132 mmol/L ↓
Potassium	3.3-5.1 mmol/L	4 mmol/L
Chloride	96-108 mmol/L	97 mmol/L
CO_2_	21-30 mmol/L	20 mmol/L ↓
Anion Gap	6-14 mmol/L	15 mmol/L ↑
Urea-Nitrogen	6-20 mg/dL	9 mg/dL
Creatinine	0.50-1.20 mg/dL	0.99 mg/dL
eGFR	≥60 mL/min/1.73m²	84 mL/min/1.73m²
Glucose	80-115 mg/dL	191 mg/dL ↑
Calcium	8.8-10.0 mg/dL	9.4 mg/dL
Bilirubin, Total	0.0-1.0 mg/dL	1.4 mg/dL↑
Bilirubin, Direct	0.0-0.3 mg/dL	0.4 mg/dL ↑
Alkaline Phosphatase	39-117 IU/L	135 IU/L ↑
AST	0-37 IU/L	42 IU/L↑
ALT	0-40 IU/L	61 IU/L ↑
Albumin	3.5-5.3 g/dL	4.2 g/dL
Protein	5.9-8.3 g/dL	8.4 g/dL ↑
INR	0.87-1.19	1.03
PT	9.4-13.0 sec	11.2 sec
Magnesium	1.3-2.1 mEq/L	1.9 mEq/L
Lipase	16-63 U/L	62 U/L
BNP	0-100 pg/mL	15 pg/mL

**Figure 1:**
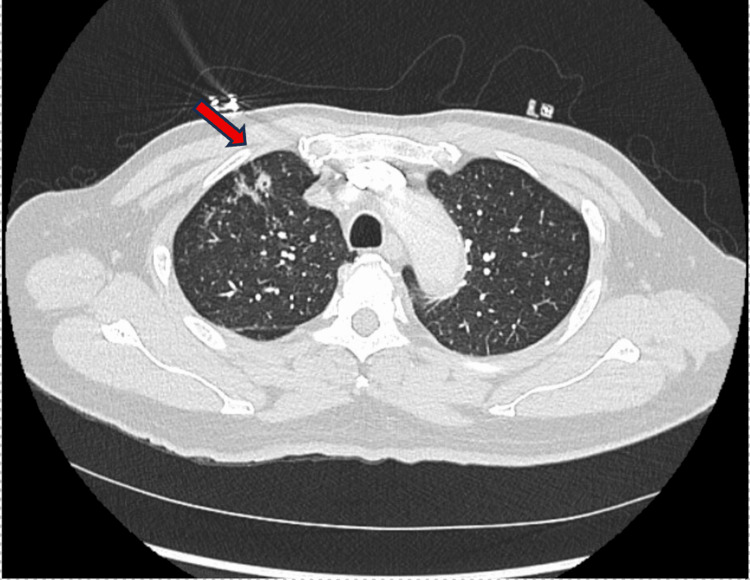
Cavitary pulmonary lesion concerning for pulmonary tuberculosis Computerized tomography angiography (CTA) chest revealed a 1 cm nodular lesion with central cavitation located in the right upper lobe of the lung (red arrow).

An initial chest X-ray demonstrated nonspecific opacities, prompting further evaluation with a CTA of the chest, which revealed a lower left lobe consolidation, a 1 cm central cavitary mass in the upper right lobe, and possible contrast outpouching in the aorta (Figure 1).

**Figure 2:**
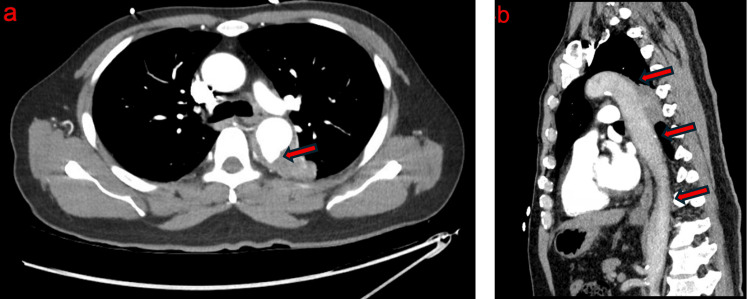
Penetrating aortic ulcer (PAU) of the descending thoracic aorta in the absence of significant atherosclerotic disease (a) Axial contrast-enhanced CT demonstrating a PAU localized to the dorsal surface of the proximal descending thoracic aorta, measuring 1.3×1.5×1.4 cm and a surrounding non-opacifying component measuring 4.2 cm (red arrow). (b) Sagittal non-contrast CT demonstrating the thoracic aorta with minimal atherosclerotic calcification and no significant plaque burden (red arrows).

Due to concern for an aortic rupture, an emergent CTA of the chest was done, which confirmed PAU in the presence of minimal atherosclerosis in the vasculature. The PAU measured 1.3×1.5×1.4 cm along the dorsal surface of the proximal descending thoracic aorta, raising clinical concern for a contained rupture of the ulcer and possible aorto-pulmonary fistula given his clinical symptoms (Figure 2).

The patient immediately underwent a thoracic endovascular aortic repair (TEVAR) to correct the aortic segment, resulting in marked improvement in his interscapular pain.

Given concerns about the other radiographic findings, in combination with the patient’s prior history of latent tuberculosis, episodes of hemoptysis, and a positive QuantiFERON-TB Gold assay (Qiagen, Venlo, The Netherlands), the medical team initiated an infectious workup for TB. The patient underwent several sputum cultures and bronchoscopy with bronchoalveolar lavage (BAL); subsequent polymerase chain reaction (PCR) testing was negative. Because he remained asymptomatic with no further episodes of hemoptysis and no microbiologic evidence of active infection at that time, he was discharged without antibiotics while cultures remained pending.

About one month later, his sputum cultures from admission grew TB, and the patient was subsequently initiated on anti-tuberculous therapy, which included rifampin, isoniazid, pyrazinamide, and ethambutol (RIPE) (Table 2).

**Table 2:** Microbiologic Testing QuantiFERON-TB Gold testing obtained at admission was positive. Initial sputum and BAL PCR testing were negative; repeat sputum PCR approximately one month after admission was positive. PCR, polymerase chain reaction; BAL, bronchoalveolar lavage.

Specimen	Test	Result
Blood	QuantiFERON-TB Gold	Positive (at admission)
Sputum	Mycobacterium tuberculosis PCR	Not detected (1 day after admission)
Bronchoalveolar lavage	Mycobacterium tuberculosis PCR	Not detected (3 days after admission)
Sputum	Mycobacterium tuberculosis PCR	Detected (~1 month after admission)

**Figure 3:**
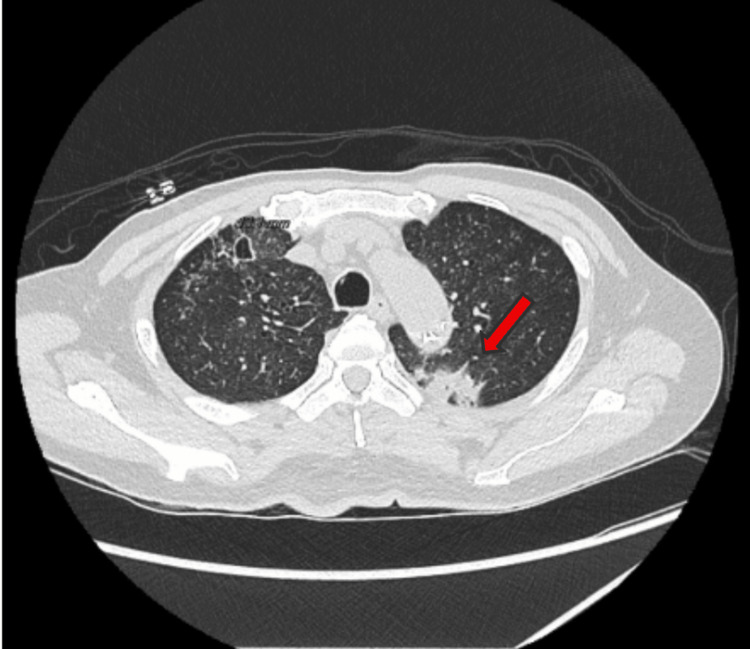
Progressive cavitary pulmonary consolidation abutting the descending thoracic aorta Axial CT of the chest at 12 weeks demonstrating a cavitary consolidative mass within the left lung adjacent to the descending thoracic aorta measuring 2.7×2.8×8.4 cm (red arrow). The lesion demonstrates evolving cavitary components compared with imaging obtained 12 weeks earlier, concerning for infection or malignancy.

Because of his poor tolerance, his regimen was downgraded to rifampin and isoniazid, and he later developed weight loss, fatigue, poor appetite, and recurrent episodes of hemoptysis, prompting his return to the hospital 12 weeks after his initial hospitalization. A repeat CTA of the chest demonstrated a mass-like airspace consolidation adjacent to the thoracic aorta, with a worsening spread of the infection (Figure 3).

He was then sequentially restarted on the RIPE regimen with careful monitoring. A positron emission tomography (PET) scan was considered because of concern for underlying malignancy; however, his positive response to treatment and the high suspicion for infection ultimately led to deferring the scan. The patient was later discharged.

**Figure 4:**
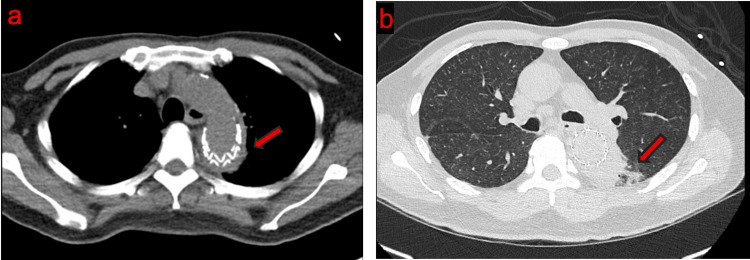
Periaortic fluid and/or aortic wall thickening with adjacent pulmonary consolidation favors the diagnosis of TA. (a) Axial contrast-enhanced CT at 20 weeks demonstrating an endovascular stent graft fully covering the previously identified PAU, with associated periaortic fluid or aortic wall thickening in the setting of minimal underlying atherosclerotic disease (red arrow). (b) Lung window demonstrating persistent consolidation in the left lung adjacent to the descending thoracic aorta (red arrow).

A routine CTA of the chest performed 20 weeks after admission demonstrated persistent periaortic fluid and aortic wall thickening around the descending thoracic aorta, with minimal atherosclerotic calcifications (Figure 4). The absence of calcified plaque along the aortic wall, together with the persistent periaortic changes and contiguous consolidation in the adjacent left lung, raised concern for an infectious etiology around the aortic segment. In light of the current radiographic findings and his clinical picture, plans were subsequently tailored toward management of TA, and a definitive reconstruction of the descending thoracic aorta was subsequently planned.

**Figure 5:**
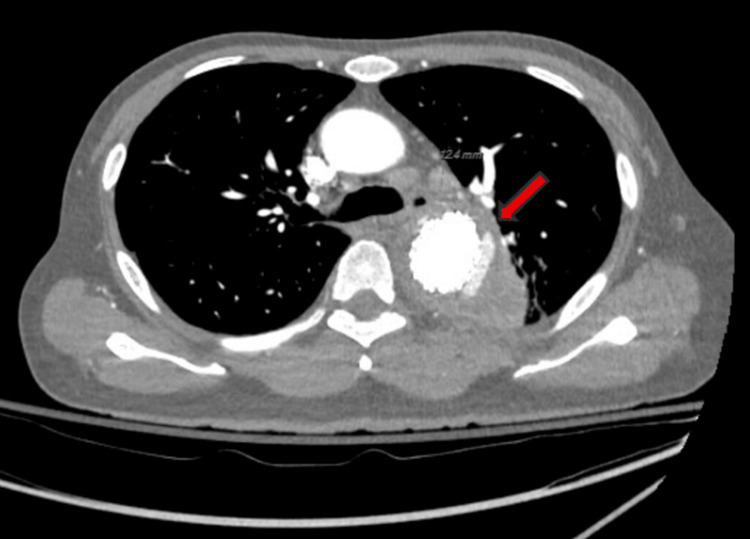
CTA chest at 24 weeks demonstrated a new contained aortic perforation with associated intramural hematoma consistent with pseudoaneurysm formation, likely secondary to tuberculous aortitis (TA) Axial contrast-enhanced CT demonstrating an endovascular stent graft within the descending thoracic aorta with a new dissection and contained perforation at the proximal landing zone measuring approximately 0.8×3.1×5.3 cm. Crescentic hyperdense blood products extending beyond the dissection flap are consistent with intramural hematoma. Associated aneurysmal dilation of the descending thoracic aorta is also noted.

At 24 weeks, the patient developed new-onset back pain, prompting readmission. Imaging revealed a new retrograde pseudoaneurysm at the mid-descending thoracic aorta, adjacent to the previous TEVAR site (Figure 5). A repeat TEVAR was performed as a temporizing measure. Once the patient was clinically stabilized, the plan was to proceed with a definitive thoracic aortic reconstruction to prevent rupture and further infectious complications.

## Discussion

This report describes a case of TB complicated by TA with a mycotic aneurysm presenting initially as PAU with minimal atherosclerotic plaque on radiography. The American College of Cardiology emphasizes that a PAU occurs in patients with advanced atherosclerosis and significant calcification, making its occurrence in a patient with minimal plaque and no traditional risk factors, as seen in our patient, highly atypical [6].

At disease onset, many prior reports of TA presented initially with constitutional features of TB, including weight loss and fatigue. A prior report suggested that of 11 patients with TA, seven presented with vascular manifestations, including claudication and asymmetric pulse readings [5]. Other reports suggested pain as the most common manifestation during pseudoaneurysm development, with one report suggesting that at least 65%-90% of cases of mycotic aneurysm secondary to TB presented with back pain [4,7].

In this case, the patient’s clinical timeline was atypical, with hemoptysis occurring initially, followed by constitutional symptoms several weeks later. He had no other clinical indicators to suggest TB other than hemoptysis at this time. The etiology of his hemoptysis could have come from either an atypical early manifestation of TB or from a PAU with possible penetration into adjacent thoracic vasculature, as seen in an earlier report [6,8]. Additionally, his episodes of hemoptysis tentatively ceased for weeks after endovascular intervention, further supporting this possibility. However, the minimal atherosclerotic burden argues against a classic PAU, raising the possibility that these findings were either coincidental or that an underlying infectious process contributed to aortic wall vulnerability and subsequent ulcer formation.

Additionally, he presented with mild laboratory abnormalities on admission. His elevated monocyte count may have been an early indicator of underlying TB. The cause of his elevated liver enzymes and mildly elevated bilirubin remains unclear but may have been reactive to the PAU or secondary to early TB, as suggested in a prior report [9].

The initial management of the PAU was driven by its depth of ~1.5 cm, which, in the setting of interscapular pain, placed the patient at high risk for aortic rupture. Even if infectious in etiology, this risk warranted immediate endovascular repair.

Radiographic follow-up 12 weeks later showed a left lower lobe consolidation adjacent to the aortic lesion, likely suggesting worsening spread of TB, given his positive sputum cultures. At this point, a PET scan was considered to evaluate for malignancy; however, low suspicion for a neoplasm and positive response to the medication led to management focused on worsening pulmonary TB.

In future cases, PET imaging may be useful beyond assessing malignancy, including identifying hypermetabolic activity in the aortic wall and evaluating for inflammatory or infectious activity, which could increase suspicion for TA. This utility is limited, however, by its lack of specificity, as increased glucose uptake can also be seen in other causes of aortitis [10,11].

Management toward TA shifted primarily based on the radiologist's report obtained 20 weeks after admission. The aortic features of TA often include dilation, stenosis, ectasia, periaortic soft-tissue changes, and inflammatory changes of the aorta, with pseudoaneurysm as the most frequently reported lesion [4]. Consistent with prior findings, the patient’s imaging showed periaortic soft-tissue changes and hyperattenuating fluid [5,12]. Furthermore, evidence of contiguous extension from a cavitary lesion in the left superior lobe, in the setting of minimal atherosclerotic plaque and positive TB cultures, suggested an infectious involvement of the aortic segment, consistent with TA.

Definitive diagnosis of TA, however, requires microbiological confirmation from aortic tissue. In clinical practice, this might not always be feasible, and even when obtained, the diagnostic yield is low. In one series, TB was identified in aortic tissue in only three of 11 patients with suspected TA, reflecting the broader challenge of diagnosing extrapulmonary TB, where mycobacterial culture sensitivity is more limited [5,13]. Although a biopsy can support the diagnosis of TA, its invasive nature means it should generally be reserved for cases in which the diagnosis remains uncertain despite clinical progression and imaging findings, or when tissue can be obtained during aortic reconstruction. The diagnosis in our case was based on a combination of multimodality imaging, clinical progression, and associated infectious findings, without tissue sampling.

The pseudoaneurysm development near the graft site at 24 weeks could have been a complication of the initial PAU; however, the underlying TB infection likely compromised the integrity of the aortic segment, leading to recurrence of his clinical presentation. This progression highlights the temporizing nature of TEVAR, rather than a definitive solution, in cases suggestive of TA and underscores the limitations of endovascular repair in infectious aortitis. In such cases, definitive management requires surgical debridement and aortic reconstruction in conjunction with appropriate antimicrobial therapy (Table 3).

**Table 3:** Timeline of Clinical Course, Diagnostic Workup, and Management Abbreviations: TEVAR, thoracic endovascular aortic repair; TB, tuberculosis; PAU, penetrating aortic ulcer; TA, tuberculous aortitis; RIPE, rifampin, isoniazid, pyrazinamide, and ethambutol

Timeline	Clinical context	Imaging/testing	Interpretation and management
Initial presentation	Interscapular pain, hemoptysis	CTA showed a focal descending thoracic aortic lesion interpreted as PAU	TEVAR performed due to acute aortic risk
4 weeks	Constitutional symptoms developed, raising concern for systemic illness	TB workup became positive	Started on RIPE regimen for the management of TB
12 weeks	Ongoing pulmonary symptoms and concern for active infection	Imaging demonstrated new cavitary/consolidative pulmonary disease adjacent to the descending thoracic aorta	Concern increased for contiguous infectious spread to the aorta rather than isolated PAU
20 weeks	Follow-up imaging to assess the progression of TB	Persistent periaortic fluid/wall thickening, minimal atherosclerosis, contiguous left lung consolidation	Diagnostic shift toward presumptive TA
24 weeks	Interscapular pain	Repeat CTA demonstrated pseudoaneurysmal change near the graft site	Reinforced limitation of TEVAR and need for definitive reconstruction

Additionally, the medical team considered other causes, such as his MVA. However, most cases of MVA are associated with acute aortic injuries rather than chronic ones. Should a chronic issue develop, prior reports have documented the formation of slow-growing pseudoaneurysms. The patient’s presentation, evolving over months in the setting of recurrent TB, makes this less likely [14].

Other possible differentials included noninfectious large-vessel vasculitis and other infectious or degenerative aortic diseases.

The treatment of TA primarily involves surgical debridement of infected aortic tissue along with treatment of the underlying tuberculous infection with a medication regimen [12]. Prior reports showed that patients with symptomatic tuberculous mycotic aneurysms of the aorta had poor outcomes without surgical intervention. In contrast, those with descending tuberculous aneurysms who underwent extensive debridement demonstrated favorable postoperative recovery and improved survival beyond three years [15]. Both in-situ reconstruction with a prosthetic graft and extra-anatomic bypass appear to afford the lowest rate of mortality in patients with tuberculous pseudoaneurysm or mycotic aneurysm [5,11,15].

Notably, discontinuation of anti-tuberculous therapy should be approached cautiously, as a prior report demonstrated fatal outcomes following endoprosthesis treatment despite 16 months of TB therapy [16].

## Conclusions

To our knowledge, no previous cases have described the coexistence of TA with a mycotic aneurysm with radiographic features showing a PAU. While histopathologic confirmation is required for a definitive diagnosis, this case remains presumptive, supported by clinical and radiographic findings consistent with TA. Clinical progression despite TEVAR reinforces the need for definitive surgical management, concurrent with antimicrobial therapy, and underscores the importance of maintaining a high index of suspicion for an infectious etiology when interpreting abnormal or evolving post-TEVAR radiographic findings in the setting of suspected infection. Future studies should focus on early recognition of infectious aortitis in the setting of worsening radiographic findings or clinical progression following TEVAR, and on evaluating its long-term outcomes after surgical management.
